# Ultra-high quality factor metallic micro-cavity based on concentric double metal-insulator-metal rings

**DOI:** 10.1038/s41598-017-15906-4

**Published:** 2017-11-15

**Authors:** Meiling Jiang, Jiwei Qi, Mingsi Zhang, Qian Sun, Jing Chen, Zongqiang Chen, Xuanyi Yu, Yudong Li, Jianguo Tian

**Affiliations:** 1 MOE Key Laboratory of Weak Light Nonlinear Photonics, Tianjin Key Laboratory of Photonics and Technology of Information Science, School of Physics, Nankai University, Tianjin, 300071 China; 2 Collaborative Innovation Center of Extreme Optics, Shanxi University, Taiyuan, Shanxi 030006 China

## Abstract

We propose and numerically investigate a novel ultra-high quality (Q) factor metallic micro-cavity based on concentric double metal-insulator-metal (MIM) rings (CDMR). In this CDMR cavity, because of the angular momentum matching, the strong coupling occurs between the same order modes of the inner and outer rings with huge resonance frequency difference. Consequently, the energy distribution between in the inner and outer rings presents enormous difference. Especially, for the quasi-in-phase CDMR modes, the energy is confined in the inner ring mainly, which suppresses the radiation loss greatly and results in ultra-narrow resonance dips and ultra-high Q factors. The full width at half-maximum (FWHM) of this CDMR cavity can be less than 2 nm and the Q factor can be higher than 300. Moreover, the character of this CDMR metallic micro-cavity can be modulated by varying the gap width between the two MIM rings. Our CDMR metallic micro-cavity provides a new perspective to design the advanced optical cavity with high Q factor and small mode volumes.

## Introduction

The optical cavity has been a subject of much investigation in recent years for its crucial functional building block for low-threshold lasers^1–3^, efficient single photon sources^4,5^, optical switches^6,7^, and optical memories^8^. Over the past decades, optical cavities with high quality (Q) factor and small mode volumes have been used to confine light into ultra-small volumes and enhance the light-matter interaction^9^. Some dielectric optical cavities, such as the whispering gallery cavities, have ultrahigh Q factor, over 10^8^. However, their geometric sizes are usually as large as several micrometers^10–12^. It is generally known that the diffraction limit impedes the dielectric optical cavities size to submicron.

In recent years, various kinds of metallic cavities based on surface plasmon polaritons (SPPs) have been investigated because the metallic micro-cavities can overcome the diffraction limit and manipulate the light into subwavelength domain^13–15^. While the metal has nonnegligible optical absorption loss and radiation loss, the metallic micro-cavity’s Q factor is generally much less than ~100^16,17^. The previous study has proved that without the optical radiation loss of the metal, the Q factor of the metallic micro-cavity can be more than 1000^18^. Therefore, the radiation loss is the bottleneck to promote the Q factor of the submicron metallic micro-cavity. Thus, different types of metallic cavity structures have been researched to promote the Q factor by decreasing the radiation loss^19–22^. Lai *et al*. proposed a plasmonic system composed of a small nanodisk with a big side-coupled-nanodisk coupled to metal-insulator-metal (MIM) bus waveguide to achieve electromagnetically induced transparency^23^. By adjusting the radius of the big nanodisk, the full width at half-maximum (FWHM) can be less than 30 nm. Chen *et al*. investigated a plasmonic refractive index sensor with high figure of merit based on a fillet cavity^24^. By varying the coupled distance between the waveguide and the fillet cavity, the FWHM can be as narrow as 12 nm. Wu *et al*. proposed a high resolution filter based on the square metallic micro-cavity^25^. Attributed to the coupled resonance in the square cavity, the Q factor can reach 212.5. Significantly, such values are far below the optimal achievable Q factor for metallic micro-cavity, just as mentioned in ref.^18^. Up to now, realizing the ultra-high Q factor micro-cavity with the small mode volumes is still a huge challenge.

In this letter, we design a novel metallic micro-cavity composed of MIM waveguide and the concentric double MIM rings (CDMR). Due to the special symmetry for the concentric double rings, the angular momentum matching condition plays an important role in the strong coupling process and enables strong coupling between the same order modes of the inner and outer rings with huge resonance frequency difference. As a result, for the CDMR modes, the energy distribution in the inner and outer rings presents an enormous difference. For the quasi-in-phase CDMR modes, the energy is mainly confined in the inner ring, which is beneficial for suppressing the energy loss of this CDMR metallic micro-cavity. Therefore, ultra-high Q factor, more than 300, and ultra-small FWHM, less than 2 nm, are obtained in our CDMR metallic micro-cavity, with a small geometric size of only 650 nm. In addition, by varying the gap width of concentric double rings, the coupling strength can be adjusted, thus the character of this CDMR metallic micro-cavity can be modulated.

## Results and Discussion

**Figure 1:**
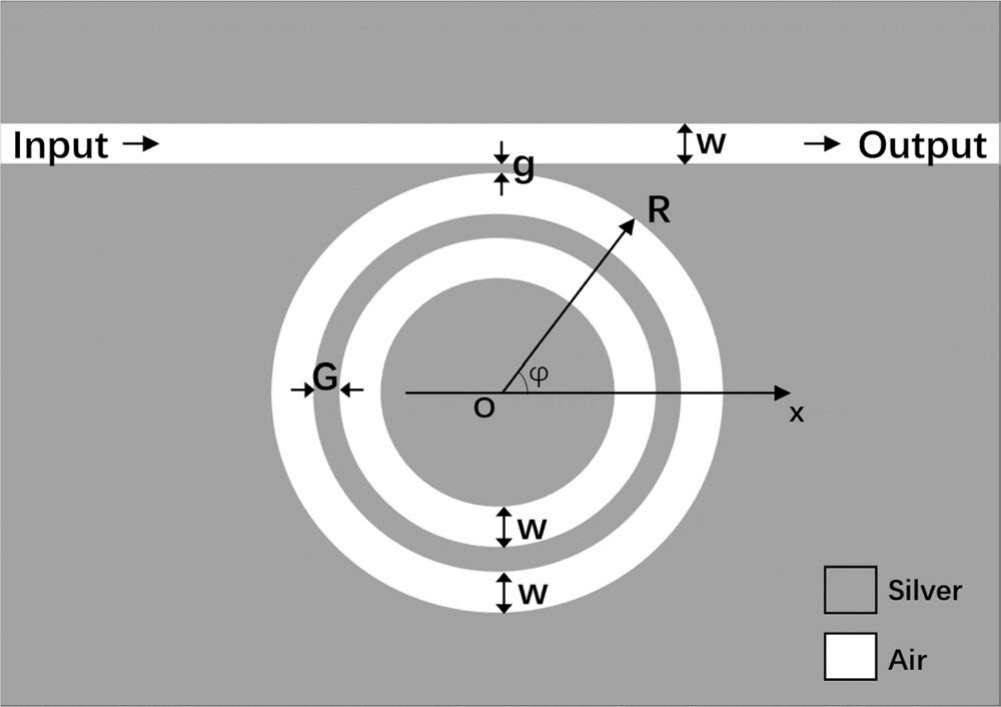
The two-dimensional schematic view of this CDMR metallic micro-cavity composed of a MIM waveguide and the CDMR.

Figure 1 shows the schematic of the two-dimensional CDMR metallic micro-cavity composed of the CDMR and the MIM waveguide. The width is *w* = 50 nm, the radius is *R* = 325 nm, and the coupling distance between the MIM waveguide and the concentric double rings is *g* = 10 nm. The gap width between the concentric double rings is *G*. The medium of the insulator is assumed to be air and the metal is set to be silver. The dielectric constant of silver is characterized by the Drude model^26^:

1 $${{\varepsilon }}_{m}=1-{{\omega }}_{p}^{2}/[{\omega }({\omega }+i\gamma )]$$

Here, *ω*_*p*_ = 1.37 × 10^16^Hz is the bulk plasma frequency, $\gamma$ = 3.21 × 10^13^ Hz is the damping frequency of the oscillations, and $\omega$ is the angular frequency of the incident electromagnetic radiation. In the following simulations, the fundamental TM mode of the plasmonic waveguide is excited by a plane wave source. The transmission spectra of the CDMR metallic micro-cavity is researched by using commercial software (COMSOL Multiphysics) of the finite-element analysis method (FEM). The transmittance is defined to be *T* = |*H*_*zout*_|^2^/|*H*_*zin*_|^2^, where |*H*_*zin*_| and |*H*_*zout*_| are the amplitudes of input and output magnetic field, respectively.

**Figure 2:**
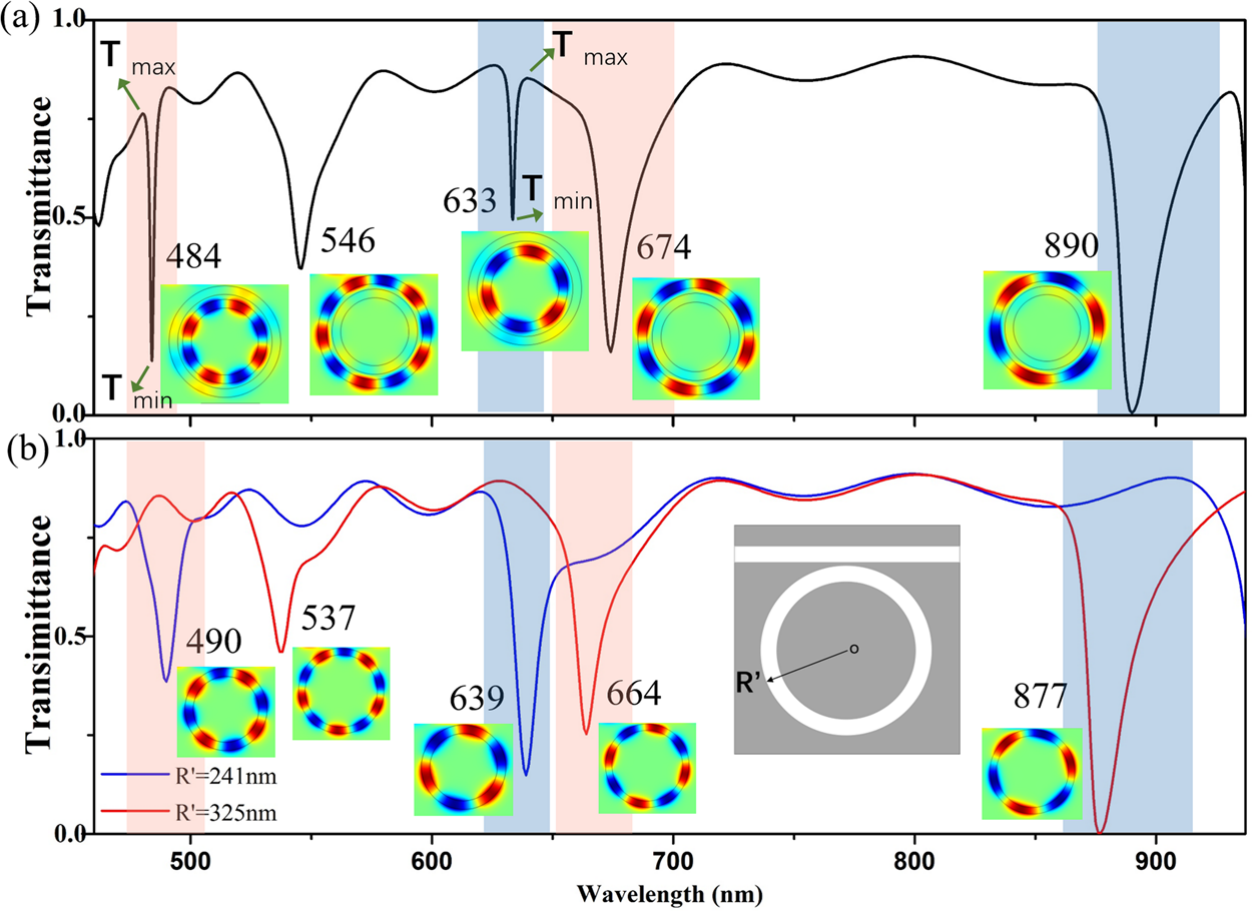
(**a**) Transmission spectrum of this CDMR metallic micro-cavity as a function of the incident wavelength with *G* = 34 nm. (**b**) Transmission spectra of each single MIM ring cavity as a function of the incident wavelength with the radius *R*′ = 241 nm and *R*′ = 325 nm respectively. The magnetic field distributions *H*_*z*_ of all these above resonance dips are also shown in this figure.

Figure 2(a) shows the transmission spectrum of the CDMR metallic micro-cavity when the gap width between the inner and outer rings is *G* = 34 nm. Two narrow resonance dips can be observed at the wavelengths of 484 nm and 633 nm respectively; three broad resonance dips locate at the wavelengths of 546 nm, 674 nm, and 890 nm respectively. The magnetic field distributions *H*_*z*_ of the resonance dips are also shown in Fig. 2(a). It is worth noting that the narrow resonance dips have small FWHM and high Q factor (defined as Q = *λ*/Δ*λ*, where *λ* is the resonance wavelength of the micro-cavity and Δ*λ* is the FWHM of transmission spectra^27^). For the narrow resonance dip located at 484 nm, the FWHM is 1.93 nm and Q factor is 250.78; for the narrow resonance dip located at 633 nm, the FWHM is 2.18 nm and Q factor is 290.37. These results demonstrate that the CDMR metallic micro-cavity we proposed does have ultra-high Q factor. Besides the Q factor, the depth of the resonance dips is also an important parameter for the practical applications. To quantitatively describe the depth of resonance dips, we define *T*_*c*_ as the contraction ratio, *T*_*c*_ = *T*_*max*_/*T*_*min*_ (*T*_*max*_ is the lower edge of the resonance dip and *T*_*min*_ is the minimum value of the resonance dip). For the narrow resonance dips located at 484 nm and 633 nm, the contraction ratios are 5.51 and 1.72, respectively. Thus, our proposed CDMR metallic micro-cavity presents ultra-high Q factor while keeping a relatively high contraction ratio which is meaningful for the practical applications.

For comparison, Fig. 2(b) shows the transmission spectra and the magnetic field distributions *H*_*z*_ of each single MIM ring that consist of the CDMR metallic micro-cavity. As shown in Fig. 2(b), the radius of the single MIM ring cavity is *R′*. When *R′* = 241 nm, which is of the same size with the inner ring of CDMR metallic micro-cavity, two resonance dips  located at the wavelengths of 490 nm and 639 nm, corresponding to the eigenmode TM_8_ and TM_6_ of the inner MIM ring cavity respectively, are present. The FWHMs are 11.86 nm and 12.67 nm respectively and the Q factors are 41.33 and 50.44, respectively. When *R*′ = 325 nm, which is of the same size with the outer ring of CDMR metallic micro-cavity, three resonance dips located at the wavelength of 537 nm, 664 nm, and 877 nm, corresponding to the eigenmode TM_10_, TM_8_ and TM_6_ mode of the outer MIM ring cavity respectively, are present. The FWHMs are 19.76 nm, 22.07 nm, and 23.18 nm, respectively and the Q factors are 27.18, 30.09, and 37.84, respectively. Clearly, the Q factor of this CDMR metallic micro-cavity we designed can be much higher than the single MIM ring cavity.

**Figure 3:**
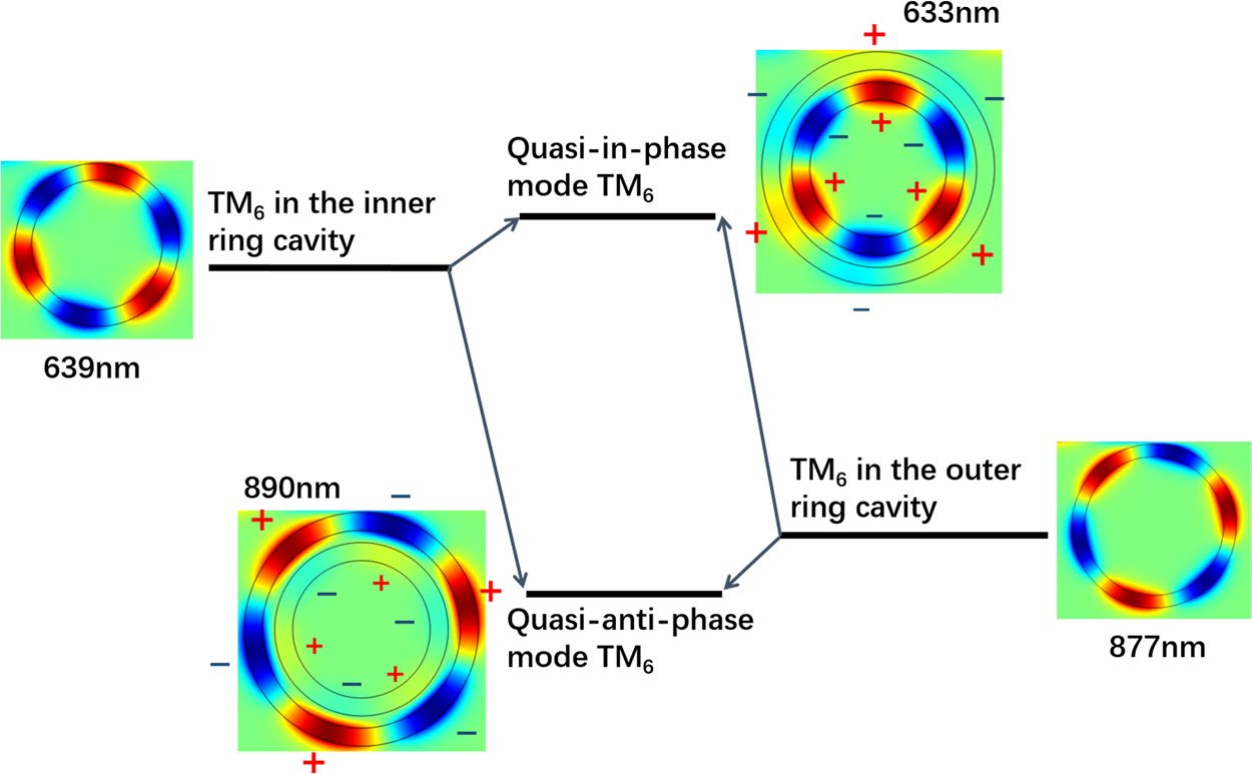
The diagram of the mode coupling between the TM_6_ of the inner and outer MIM rings in the CDMR metallic micro-cavity. The magnetic field distributions *H*_*z*_ of all these above modes and the phase distributions of the quasi-in-phase and quasi-anti-phase CDMR modes are also shown in this figure.

To explain the mechanism of the ultra-high Q factor formation, coupled mode theory^28^ is employed in this letter. As is well known, the propagation constants of the coupled two parallel waveguides need to be nearly identical to guarantee coupling effectively^29^. Analogously, in our CDMR metallic micro-cavity, the electromagnetic wave in the inner and outer rings couples with each other for a considerable long length. Considering the special symmetry of this CDMR structure, angular momentum matching is needed for the effective coupling between the electromagnetic waves propagating in the inner and outer rings. Only the same order modes of the inner and outer rings can have the fixed phase difference of 0 or *π* at any angle *φ*. The phase difference of the different order modes of the inner and outer rings changes with *φ*. This means that only the same order modes of the inner and outer rings can meet the angular momentum matching condition while the different order modes can’t. Thus, the angular momentum matching condition makes the strong coupling more inclined to take place between the same order modes between the inner and outer rings, though large resonance frequency difference exists between them as shown in our results. As shown in Fig. 2, the same order modes for the inner and outer rings of the CDMR metallic micro-cavity with large resonance frequency difference couple with each other and form the quasi-in-phase and quasi-anti-phase CDMR modes. Specifically, quasi-in-phase TM8, TM6 CDMR modes locate at 484 nm and 633 nm, respectively; quasi-anti-phase TM10, TM8, TM6 CDMR modes locate at 546 nm, 674 nm, and 890 nm, respectively. As an example, the diagram of mode coupling between the TM6 modes of the inner and outer rings of the CDMR metallic micro-cavity is shown in Fig. 3. As shown in Figs 2 and 3, the narrow resonance dips induced by the quasi-in-phase CDMR modes close to the inner ring mode and have a slight blue-shift; the broad resonance dips induced by the quasi-anti-phase CDMR modes close to the outer ring mode and have a slight red-shift. Just because of the large resonance frequency difference between the coupled same order modes, the quasi-in-phase CDMR modes are close to the inner ring mode, far away from the outer ring mode; the quasi-anti-phase CDMR modes are close to the outer ring mode, far away from the inner ring mode. As a result, the energy of electromagnetic wave mainly distributes in the inner MIM ring for the quasi-in-phase CDMR modes and mainly distributes in the outer MIM ring for the quasi-anti-phase CDMR modes, as shown in Fig. 2. The special energy distribution of the quasi-in-phase CDMR modes, the energy mainly distributed in the inner MIM ring cavity, suppresses the energy loss from the CDMR metallic micro-cavity to the MIM waveguide and is beneficial for energy storage. Therefore, the resonance dips induced by the quasi-in-phase CDMR modes are very narrow and the Q factors are very high. On the contrary, for the quasi-anti-phase CDMR modes, the energy mainly distributes in the outer MIM ring cavity, which is unsuitable for energy storage. Consequently, the resonance dips induced by the quasi-anti-phase CDMR modes are broad and the Q factors are low. In brief, in our proposed CDMR metallic micro-cavity, the angular momentum matching condition plays a dominant role in strong coupling between the inner and the outer rings and enables the strong coupling between the same order modes with huge resonance frequency difference. Then, the large resonance frequency difference between the coupled modes results in an enormous difference between the energy distribution in the inner and outer rings for the CDMR modes. For the quasi-in-phase CDMR modes, the energy of electromagnetic wave confines in the inner MIM ring which is benefit to suppress the loss and also leads to ultra-small FWHM and ultra-high Q factor.

**Figure 4:**
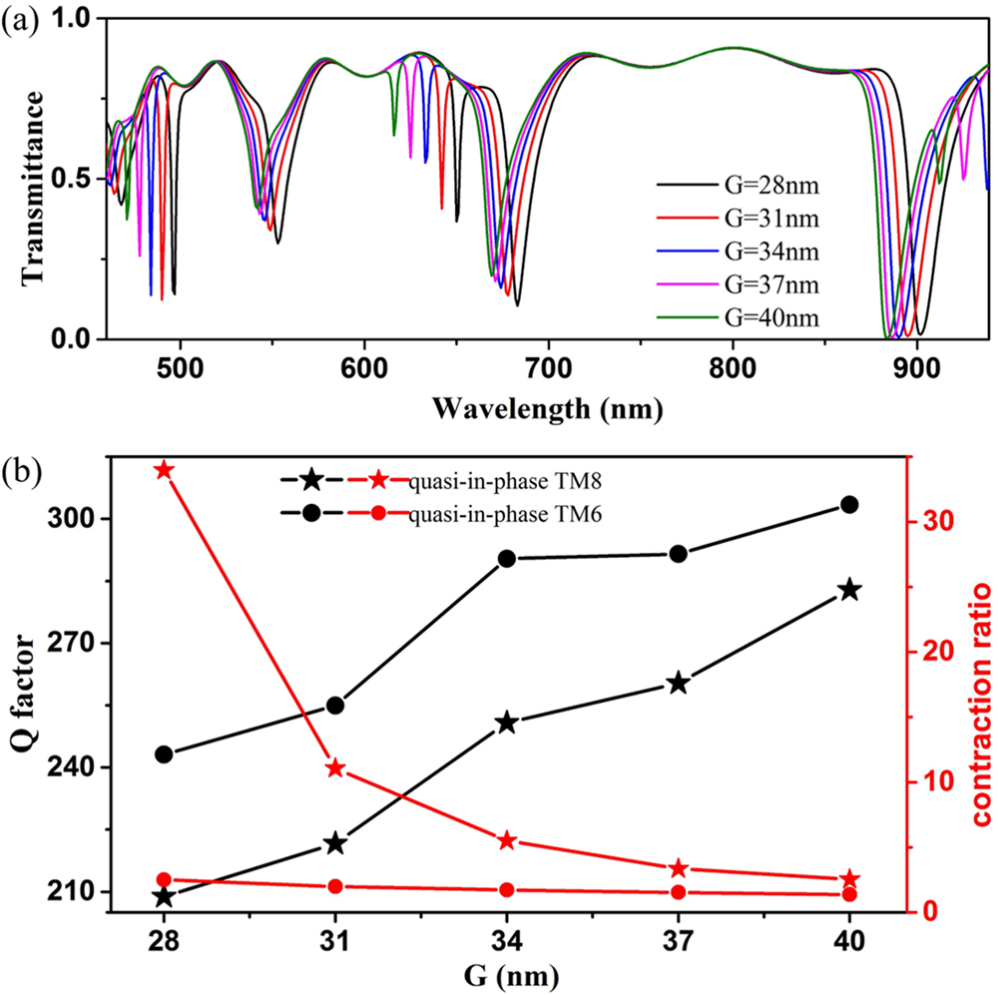
(**a**) The transmission spectra of the CDMR metallic micro-cavity with coupling distance *G* increasing from 28 nm to 40 nm with step length of 3 nm. (**b**) The Q factors and transmission contraction ratios of quasi-in-phase mode TM_6_ and TM_8_ respectively versus coupling width *G*.

The coupling strength plays an important role in the formation of ultra-small FWHM and ultra-high Q factor for the coupled mode theory. Therefore, the character of the resonance dips can be tuned by changing the gap width *G* between the inner and the outer MIM rings. To keep the size of this metallic micro-cavity unchanged, in our simulation, we keep the radius of the outer MIM ring constant as *R* = 325 nm and decrease the radius of the inner MIM ring. The results are shown in Fig. 4(a), the transmission spectra of this CDMR metallic micro-cavity as *G* increases from 28 nm to 40 nm with the step length of 3 nm. It is observed that the increase of *G* leads to a large blue-shift of the quasi-in-phase CDMR modes and a small blue-shift of the quasi-anti-phase CDMR modes. The Q factors and contraction ratios of the quasi-in-phase TM_6_ and TM_8_ CDMR modes with the varying *G* are calculated and depicted in Fig. 4(b). For the quasi-in-phase TM_6_ and TM_8_ CDMR modes, the Q factors increase and the contraction ratios decrease with the increase of *G*. With the increase of *G*, the coupling strength between the inner and outer rings of the CDMR metallic micro-cavity becomes weaker. And then, less field energy of quasi-in-phase CDMR modes, which mainly confined in the inner MIM ring, can be coupled to the outer MIM ring and the MIM waveguide. The energy can be confined in the inner ring effectively, which suppresses the radiation loss to the outer ring and waveguide greatly. Thus, the increase of *G* results in a decrease of the coupling loss of the quasi-in-phase modes of the CDMR metallic micro-cavity and consequently results in increase of the Q factors for quasi-in-phase CDMR modes. As shown in Fig. 4(b), when the coupling width *G* reaches 40, the Q factors of quasi-in-phase TM_8_ and TM_6_ CDMR modes are 282.89 and 303.45 respectively. However, with the increase of G, the contraction ratio becomes smaller. This is caused by that littler energy can be coupled into the inner ring of the CDMR metallic micro-cavity, when the coupling strength between the inner and outer rings is weaker. When the coupling width G = 40 nm, the contraction ratios of quasi-in-phase TM_8_ and TM_6_ CDMR modes are 2.54 and 1.37 respectively. Therefore, there is a tradeoff between the high Q factor and the high contraction ratio.

## Conclusion

In summary, high Q factor and small mode volumes CDMR metallic micro-cavity is proposed and investigated in detail. In our CDMR metallic micro-cavity, due to the special symmetry for the CDMR, the angular momentum matching condition plays a dominant role in the strong coupling process, which makes the strong coupling take place between the same order modes of the inner and outer rings of the metallic micro-cavity with a huge resonance frequency difference. Then, for the CDMR modes, enormous difference between the energy distribution in the inner and outer rings presents. For the quasi-in-phase CDMR modes, the energy distributes in the inner MIM ring mainly and the energy loss from the CDMR metallic micro-cavity is suppressed effectively. Therefore, the Q factor of the CDMR metallic micro-cavity can be ultra-high, more than 300, and the FWHM can be ultra-small, less than 2 nm, with a very small cavity size, about 650 nm. Furthermore, we show that when the ultra-high Q factor is obtained, the transmission contraction ratio of the resonance dips is keep with a large value, which is important for practical applications. Moreover, the character of this CDMR metallic micro-cavity can be modulated by varying the gap width between the concentric double rings. The feature of this CDMR metallic micro-cavity will greatly promote the development of optical cavity towards the higher Q factor and smaller mode volumes.
